# The T Cell Receptor: Molecular Sensor, Therapeutic Mediator and Probabilistic Driver of Adaptive Immunity

**DOI:** 10.1111/imr.70136

**Published:** 2026-06-04

**Authors:** Kilian Schober

**Affiliations:** ^1^ Mikrobiologisches Institut–Klinische Mikrobiologie, Immunologie und Hygiene Universitätsklinikum Erlangen, Friedrich‐Alexander‐Universität Erlangen‐Nürnberg Erlangen Germany; ^2^ FAU Profile Center Immunomedicine FAU Erlangen‐Nürnberg Erlangen Germany

## Abstract

Advances in high‐throughput sequencing, single‐cell profiling, and genome engineering have transformed the study of T cell receptors (TCRs), enabling the identification and functional interrogation of antigen‐specific repertoires at an unprecedented scale. This review discusses how recent methodological developments—including high‐dimensional TCR discovery strategies, physiological receptor engineering, and longitudinal in vivo analyses—have reshaped our understanding of TCR‐driven immune responses. Recruitment into immune responses originates from diverse naïve precursor pools and results in polyclonal populations in which multiple clonotypes contribute to antigen recognition. Within such populations, receptor properties, such as TCR avidity, influence the likelihood of recruitment, expansion, and persistence. However, the impact of these parameters depends strongly on biological context, including antigen availability, cellular competition, and tissue environment. Physiological engineering approaches, such as orthotopic TCR replacement, now enable causal interrogation of receptor function while preserving endogenous regulatory control. Together with advances in spatial and longitudinal profiling of human immune responses, these approaches allow increasingly precise analyses of connections between TCR identity and T cell fate. Integrating insights across antigen discovery, receptor engineering, and in vivo dynamics suggests that TCR biology is shaped by the interplay of receptor sequence, regulatory context, and tissue environment across time. Understanding these relationships will be essential for interpreting immune responses and for guiding the rational design of T cell‐based immunotherapies.

## 1 Introduction

The T cell receptor (TCR) is a defining molecular feature of adaptive cellular immunity. By recognizing peptide fragments presented by major histocompatibility complex (MHC) molecules, TCRs enable T cells to survey intracellular protein content across nucleated cells—in contrast to antibodies or chimeric antigen receptors (CARs), which are largely restricted to extracellular or cell‐surface targets. This mode of antigen recognition underlies T cell‐mediated immunity against infections, malignant transformation, and a wide range of autoimmune and inflammatory diseases.

Beyond conferring antigen specificity, TCRs influence how T cells respond once antigen is encountered. Differences in binding properties of TCR‐peptide–MHC (TCR‐pMHC) interactions shape activation, recruitment into immune responses, clonal expansion, and differentiation over time. As a result, TCRs do not operate as simple binary switches, but encode quantitative information that affects and fine‐tunes downstream signaling and cellular behavior. How such receptor‐level information is translated into effective and robust immune responses remains an open question in basic T cell immunology and immunotherapy.

Historically, TCR biology has been studied from partially disconnected perspectives. Structural and biophysical approaches have focused on receptor‐ligand interactions, whereas immunological studies have emphasized the phenotypes and dynamics of antigen‐specific T cell populations in vivo. While both approaches have yielded fundamental insights, they have often been difficult to reconcile, particularly in human systems. As a consequence, it remains difficult to link defined properties of individual TCRs to population‐level immune outcomes.

In recent years, this situation has begun to change. Advances in single‐cell technologies now allow paired (αβ or δγ) TCR identification together with detailed phenotypic characterization, while novel high‐throughput methods enable systematic interrogation of antigen specificity at unprecedented scale. In parallel, TCR engineering makes it possible to test causal relationships between receptor identity and T cell behavior, while spatially resolved transcriptomic approaches provide new insight into how immune responses unfold within tissues. Together, these developments enable the investigation of TCR biology across molecular, cellular, and population scales with increasing precision.

At the same time, the success of CAR T cell therapies has provided a compelling proof of principle that adoptive cell therapy with engineered antigen receptors can achieve sustained clinical benefit [1]. Importantly, the clinical efficacy of CAR T cells—most prominently in CD19‐targeted therapies—has shown that therapeutic success depends as much on the biology of the target antigen as on receptor design. This realization has refocused attention on TCRs, which offer access to the intracellular antigen space and thereby a vastly expanded set of potential therapeutic targets, but also impose distinct biological constraints [2].

These developments have brought TCR engineering into sharper focus, both as a therapeutic strategy and as an experimental approach. While engineered TCRs hold promise for cancer and chronic infections, their broader application is limited by human leukocyte antigen (HLA) restriction, challenges in epitope identification, and incomplete understanding of how engineered receptors are regulated and maintained in vivo (hereafter, HLA refers to the human system and MHC to murine or species‐independent systems). Addressing these limitations requires not only improved engineering strategies but also deeper insight into the principles that govern TCR‐driven immune responses.

Much of our current understanding of TCR biology has been shaped by experimental work in mouse models, which have been indispensable for establishing foundational concepts in T cell immunology. These models enabled mechanistic dissection of TCR signaling, clonal dynamics, and fate decisions that would not have been possible otherwise. However, translating these insights to human immunity remains a central challenge [3]. The integration of high‐dimensional human data with principles derived from experimental models offers a path towards a more comprehensive understanding of TCR‐driven human immunity.

In this review, which is biased towards my own work, I synthesize recent progress in the study of αβ TCR biology with a focus on three interrelated questions. First, how can antigen‐specific TCRs be identified and prioritized under conditions of extreme TCR‐pMHC combinatorial diversity? Second, how can TCRs be engineered in a manner that enables causal inference while preserving physiological regulation? Third, how do TCR‐driven immune responses evolve in vivo over time and space to give rise to effective or dysfunctional immunity? By integrating insights from high‐dimensional identification strategies, physiological engineering approaches, and in vivo fate analysis, this review seeks to clarify how TCRs shape adaptive immune responses. While there will be a particular emphasis on human immunity, preclinical model systems remain critically important.

*This review places my work of the past decade within the broader context of TCR research and therefore inevitably reflects a personal perspective. The studies highlighted here represent individual contributions within a much larger collective effort to understand TCR biology*.

## 2 Identifying Antigen‐Specific T Cell Receptors Under Extreme Diversity

### 2.1 Historical and Technological Foundations

The identification of antigen‐specific TCRs has long been an experimental bottleneck in T cell immunology. Early approaches relied on functional readouts at the population level, such as proliferation or cytokine production after antigen stimulation, which provided indirect evidence of specificity but did not allow direct linkage to individual TCR sequences. A major conceptual advance came with the introduction of pMHC multimer reagents, which enabled direct visualization and isolation of antigen‐specific T cells based on pMHC‐TCR binding [4, 5]. These reagents established a foundation for antigen‐specific immunology by allowing ex vivo identification of rare T cell populations without prior in vitro expansion.

Initial efforts to recover TCR sequences from antigen‐specific T cells were technically demanding and low throughput. Single‐cell PCR‐based approaches required physical isolation of individual cells into wells, followed by separate amplification of TCR α and β chains, often with limited efficiency and incomplete pairing [6, 7, 8]. Although these methods enabled the first direct connections between antigen specificity and TCR sequence, they were not scalable and were restricted to small numbers of cells and receptors.

The past decade has seen a fundamental shift that was driven by the advent of single‐cell RNA sequencing technologies. These approaches allow the parallel capture of paired TCR chains together with transcriptomic profiles from thousands to tens of thousands of individual T cells in a single experiment [9, 10]. As a result, TCR sequence information can now be directly linked to cellular phenotype, activation state, and differentiation status. Additionally, statistical inference of data obtained through bulk TCR α and β chain sequencing—as done in TIRTL‐seq [11]—allows pairing of even more cells to a lower price per cell, albeit at the cost of not being compatible with phenotypic profiling. This technological convergence transformed TCR analysis from a low‐throughput, candidate‐driven endeavor, into a high‐dimensional, discovery‐oriented field.

In parallel, pMHC multimer technologies have continued to evolve. Reversible and modular multimer formats increased experimental flexibility and preserved cellular viability, enabling downstream functional and transcriptional analyses [12, 13, 14, 15]. The introduction of DNA‐barcoded pMHC multimers together with next‐generation sequencing further expanded the scale at which antigen specificity could be interrogated, allowing hundreds to thousands of epitopes to be tested simultaneously in a single reaction [16, 17]. These developments substantially increased the dimensionality of antigen‐specific T cell profiling and enabled systematic interrogation of TCR‐epitope relationships.

More recently, several high‐throughput platforms have emerged for TCR deorphanization with up to genome‐wide unbiased epitope discovery scale for individual TCRs [18]. Particularly intriguing are viral display‐based approaches for presenting pMHC complexes [19, 20, 21]. By leveraging pseudotyped viral particles, these methods allow the efficient and versatile generation of highly diverse pMHC libraries that can be interrogated in pooled formats. Together with advances in single‐cell sequencing, such technologies now permit the analysis of TCR specificity at a scale and combinatorial resolution that was inconceivable only a decade ago.

Collectively, these historical and technological developments have shifted the bottleneck in TCR research. While the identification of antigen‐specific T cells and their receptors was once limited by experimental feasibility, it is now increasingly constrained by combinatorial complexity and data availability. The following sections build on this foundation to discuss how antigen‐specific TCRs can be prioritized under extreme diversity and how methodological advances have reshaped the identification of biologically or clinically relevant TCRs in complex human immune responses.

### 2.2 Combinatorial Complexity of TCR‐Epitope Pairs

**FIGURE 1:**
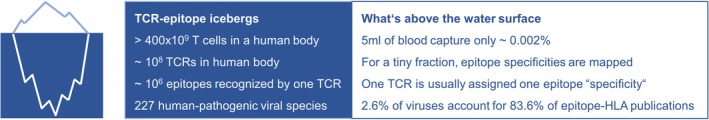
TCR‐epitope icebergs. Due to the diversity of TCRs, epitopes, and combinations thereof, significant knowledge gaps exist. What we know is often just “the tip of the iceberg”. This concerns, for example, the fraction of T cells captured by a typical blood sample [22]; the number of epitope‐mapped TCRs in relation to the total number of TCRs in a given organism [23]; the number of epitopes that can be recognized by a given TCR [24]; or the number of viral pathogens for which robust knowledge on epitopes exist (numbers are derived from www.imdb.org; for the sake of simplicity, the numbers for non‐viral pathogens are not indicated).

**FIGURE 2:**
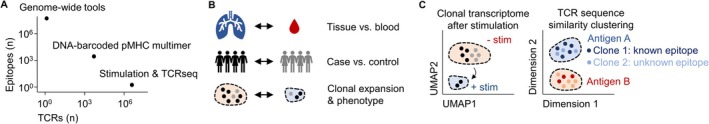
TCR‐epitope mapping strategies. (A) Different tools have different capabilities of mapping many epitopes for few TCRs or vice versa. The indicated strategies are just three examples out of many, to illustrate that combinatorial complexity of TCRs versus epitopes provides a limiting factor [18]. (B) Antigen‐agnostic strategies to identify clonotypes that are relevant for a given setting (e.g., a disease). (C) Antigen‐driven strategies may (next to strategies shown in A) employ “reverse phenotyping” [26] or TCR sequence similarity‐based metrics [27, 28].

Despite substantial advances in the experimental identification of antigen‐specific TCRs, a fundamental challenge remains: the extreme combinatorial complexity of the TCR‐epitope interaction space (Figure 1). Each human is estimated to harbor about 100 million distinct TCRs [23], while the universe of potential pHLA ligands spans a similarly vast space shaped by antigen diversity, antigen processing, and HLA polymorphism [24]. For a single MHC molecule, an epitope space of 10^6^ different peptides has been estimated [25]. Even with high‐throughput technologies, only a small fraction of all possible TCR‐epitope combinations can be interrogated experimentally. As a ballpark estimate, a combinatorial complexity of 10^5^–10^7^ unique TCR‐pMHC interactions appears to be a current experimental ceiling within one analysis. In other words, one may screen 1 TCR against 1 million epitopes, 1 thousand TCRs against 1 thousand epitopes, or 1 million TCRs against a single epitope (Figure 2A). In practice, this means that experimental approaches must operate under constraints that preclude exhaustive screening. Even technologies that allow the parallel testing of hundreds or thousands of epitopes remain orders of magnitude below the theoretical complexity of the TCR‐epitope space.

Consequently, for the vast majority of TCRs in an individual, the antigen specificities are unknown. One reason for this is that human TCR analyses are typically based on peripheral blood samples since tissue accessibility is limited. If one assumes a total blood volume of 5 L, a typical sample of 5–50 mL of blood is equivalent to 0.1%–1% of the total blood volume. As 98% of immune cells reside outside the blood [22], a typical experimental sample would therefore capture 0.002%–0.02% of T cells from the entire organism (Figure 1). This simplified calculation illustrates the magnitude of sampling bias. T cell immunologists are aware of this bias yet often underestimate its implications. Immunologists may learn therefore from the field of ecology, which is fully aware of the “unseen species problem” [29].

Importantly, combinatorial complexity also limits purely computational solutions. While sequence‐based similarity measures and machine learning approaches have made substantial progress in identifying related TCRs and predicting specificity within known epitope families, they remain constrained by the availability and diversity of training data [30]. Thus, the challenge of combinatorial complexity is not only experimental but also informational, reflecting gaps in the underlying data landscape.

Together, these considerations highlight that the key bottleneck in identifying antigen‐specific TCRs is no longer the ability to measure receptor sequences or associated cellular phenotypes, but the ability to reduce the search space in a biologically meaningful way. The practical question therefore shifts from *can we measure TCRs* to *which TCRs are worth measuring in the first place*. The following sections discuss biological and experimental strategies that address this problem by prioritizing candidate TCRs before direct interrogation of antigen specificity.

### 2.3 Antigen‐Agnostic Prioritization Strategies

Given the infeasibility of exhaustively interrogating the full TCR‐epitope space, prioritization becomes essential. Thereby, antigen‐agnostic strategies do not directly establish specificity, but instead enrich for TCRs that are more likely to be biologically relevant in a given disease context (Figure 2B). Conceptually, they shift the problem from *what a TCR recognizes* to *where, when, and in which cells the TCR is found*.

One commonly used criterion is *tissue enrichment*. TCR clonotypes that are overrepresented in diseased tissue compared with peripheral blood are more likely to have encountered antigen locally and to participate in tissue‐specific immune responses. This principle has been applied across diverse settings, including tumors, sites of persistent infection and inflamed organs—for example, cerebrospinal fluid (CSF) in multiple sclerosis [31]. As with other criteria, tissue enrichment does not imply antigen specificity per se. For example, many TCRs found in human tumors recognize viral antigens [32, 33, 34]. However, tissue enrichment does serve as a probabilistic filter that reduces the candidate space.

A second strategy relies on *case–control enrichment*. By comparing TCR repertoires between patients and matched controls, clonotypes that are selectively expanded or recurrent in disease cohorts can be identified. Harlan Robins and the company Adaptive Biotechnologies first demonstrated this approach for Cytomegalovirus (CMV) in a landmark study [35], exploiting the fact that antigen‐driven immune responses often leave reproducible imprints at the repertoire level across individuals. While this strategy is powerful at the population level, it typically lacks resolution at the level of individual epitopes and requires large, well‐curated cohorts to avoid confounding effects.

*Clonal expansion* represents a third, orthogonal prioritization criterion. Antigen encounter frequently leads to proliferation of responding T cells, resulting in skewed clonal size distributions within the repertoire. Expanded clonotypes are therefore enriched for antigen‐experienced T cells, although expansion alone does not distinguish between protective, bystander, or pathogenic responses [32, 36]. Moreover, expansion can reflect past rather than ongoing immune activity, underscoring the importance of integrating clonal information with additional context.

Finally, *phenotypic association* provides an additional layer of prioritization. Antigen‐experienced T cells often acquire characteristic transcriptional or surface marker profiles that reflect activation, differentiation, or functional specialization. By linking TCR sequences to such phenotypes through single‐cell approaches, clonotypes associated with specific cellular states can be enriched. Modern computational tools allow joint embedding of TCR sequence and cellular phenotype [37, 38, 39].

Individually, each of these criteria provides only limited information. However, when combined, they offer a powerful means of reducing the effective search space for antigen‐specific TCRs by several orders of magnitude. In practice, meaningful prioritization often requires combining at least two of these criteria. These strategies are broadly applicable across disease categories, including infections, autoimmunity, and cancer, and do not depend on prior knowledge of the relevant antigens.

### 2.4 TCR Repertoires for Disease Diagnostics and Immune History Reconstruction

Beyond mechanistic insight, antigen‐agnostic TCR repertoire analysis opens a fundamentally new avenue for immune diagnostics. Immune exposures leave durable and partially reproducible imprints on TCR repertoires across individuals, reflected in the enrichment, co‐occurrence, or depletion of specific clonotypes or sequence motifs [35, 40, 41, 42]. Rather than requiring explicit identification of cognate epitopes, diagnostic approaches can exploit these repertoire‐level signatures, treating TCR sequences as integrative biomarkers of immune history.

The conceptual leap of this approach is substantial. Conventional serological assays are inherently epitope‐specific and pathogen‐focused: each test is designed to detect antibodies against a defined antigen and provides information limited to that exposure. In contrast, a single TCR repertoire sequencing experiment has the potential to capture a broad spectrum of immune experiences simultaneously. This resembles the VirScan approach that identifies antibody responses across the human virome [43]. However, TCR repertoire‐based analysis instead remains antigen‐agnostic, which enables detection of immune responses to any kind of pathogen or also non‐infectious immune exposures like autoimmune diseases [40]. One assay could therefore report on prior infections, vaccinations, and immune perturbations accumulated over a lifetime, without requiring prior specification of which disease to interrogate. An intriguing implication is the temporal flexibility of such data: as training datasets expand, TCR repertoires generated years or even decades earlier could, in principle, be reanalyzed to infer immune exposures that were not identifiable at the time of sampling. In this sense, TCR repertoire data may function as a biological record whose informational value increases over time.

At the same time, the power of repertoire‐scale diagnostics necessitates careful consideration of clinical responsibility. High‐dimensional immune profiling inevitably generates findings of uncertain significance, raising the risk of overinterpretation, overdiagnosis, and potentially unnecessary intervention. Without clear clinical frameworks, broad immune readouts may impose an additional burden on patients and healthcare systems rather than providing actionable benefit. Responsible implementation therefore requires stringent validation, well‐defined use cases, and close integration with clinical decision‐making pathways.

For these reasons, the most plausible clinical applications are scenarios in which a broad assessment of immune status is already clinically sensible (for example during early pregnancy or after hematopoietic stem cell transplantation), rather than indiscriminate population‐wide screening. In addition to research applications, one particularly promising area is the diagnosis and stratification of autoimmune diseases, where T cell‐mediated pathology plays a central role [44]. For past pathogen exposures, conventional serological testing often provides a precise binary readout (e.g., previous EBV exposure: yes or no). In contrast, diagnosis of autoimmune diseases often results from combining tedious and expensive workflows, spanning patient history and physical examination, imaging, and auto‐antibody testing. Indeed, early studies have demonstrated the feasibility of using TCR repertoire signatures to identify autoimmune conditions such as primary sclerosing cholangitis, rheumatoid arthritis, systemic lupus erythematosus or type 1 diabetes [40, 45, 46, 47], highlighting contexts in which repertoire‐based diagnostics may offer information that complements or refines existing assays.

As reference datasets grow in size, diversity, and clinical annotation, TCR‐based diagnostics may mature into a powerful addition to the diagnostic toolbox. Realizing this potential will depend not only on technical and analytical advances, but also on careful stewardship of data and thoughtful integration into clinical practice. When applied judiciously, repertoire analysis offers the prospect of a unified, systems‐level view of immune history that is fundamentally distinct from—and in specific contexts complementary to—conventional antibody‐based diagnostics. Finally, clonotypes identified as disease‐associated through unbiased diagnostic workflows may represent the most rational starting point for further mechanistic interrogation (instead of the approaches listed in 2.3). Identifying the antigen specificity of such TCRs may provide direct insight into disease pathophysiology in the most effective manner.

### 2.5 Antigen‐Driven Identification Without Epitope Resolution

While antigen‐agnostic strategies provide powerful means to prioritize candidate TCRs, many experimental settings allow controlled antigen exposure even without requiring prior knowledge of the precise epitope. In such cases, T cells can be stimulated with complex antigen sources—such as whole proteins, peptide pools, pathogen‐derived lysates, or tumor cells—and responding cells can be identified based on functional or transcriptional changes (Figure 2C). These approaches occupy an intermediate position between direct epitope‐resolved specificity mapping and purely antigen‐agnostic prioritization.

A commonly used strategy involves short‐term in vitro stimulation followed by detection of activation‐induced markers or cytokine production. This enables enrichment of antigen‐responsive T cells and subsequent analysis of their TCR repertoires. Such approaches have been particularly valuable for studying CD4^+^ T cell responses, for which the generation of stable pMHC class II multimers is more challenging and antigen specificity is often less well defined than for CD8^+^ T cells [48, 49].

Antigen‐driven stimulation approaches offer several advantages (in this context, “antigen‐driven” refers to techniques that identify T cells based on functional or transcriptional reactivity to antigen exposure, rather than direct binding to defined pMHC complexes). They do not require prior knowledge of restricting HLA alleles or minimal epitopes; they are scalable to complex antigens, and they can be applied across diverse disease contexts [50]. At the same time, they introduce important limitations. Stimulation perturbs the transcriptional and functional state of responding T cells, complicating interpretation of their baseline phenotype [26]. Moreover, reliance on predefined activation markers risks systematically missing subsets of responding cells that do not conform to canonical activation patterns.

These considerations highlight a central trade‐off inherent to antigen‐driven identification without epitope resolution: while such approaches provide a direct link between antigen exposure and TCR identity, they do so at the cost of altering the very cellular states one may wish to study. Addressing this tension requires strategies that retain the advantages of antigen‐driven enrichment while preserving access to the pre‐stimulation phenotypic landscape of responding T cells.

### 2.6 Reverse Phenotyping

Antigen‐driven stimulation enriches for reactive T cells but perturbs their transcriptional and functional state [51]. As a result, information about the baseline phenotype that preceded antigen encounter is partially or fully obscured. We developed “reverse phenotyping” to address this limitation by separating identification of antigen reactivity from characterization of unperturbed cellular states [26].

In reverse phenotyping, T cells are analyzed in parallel under stimulated and unstimulated conditions, followed by single‐cell transcriptomics and TCR sequencing. Antigen‐reactive clonotypes are identified based on antigen‐induced transcriptional changes observed upon stimulation (Figure 2C). Once reactive clonotypes are identified, their TCR is used as a molecular barcode and phenotypic properties can be traced back to the unstimulated condition, thereby revealing the cellular states these T cells occupied prior to antigen exposure. This strategy enables identification of antigen‐reactive T cells without relying on predefined activation markers and without assuming a priori which phenotypic states are relevant.

A key strength of this approach is its unbiased nature. Because antigen‐reactive cells are identified based on global transcriptional shifts rather than enrichment for specific surface markers or cytokines, reverse phenotyping can reveal unexpected patterns of antigen responsiveness and uncover unperturbed phenotypic states that would be missed by conventional activation‐induced marker assays. For example, after SARS‐CoV‐2 infection [26] and vaccination [52], spike‐specific CD4^+^ T cells exhibited a more Th1‐like phenotype following ex vivo antigen stimulation than in the unstimulated state in which they appear more Th‐neutral—not only with regard to effector molecules like *IFNG*, but also in terms of transcription factors like *TBX21*. These data can inspire the discussion of whether cells should be assessed based on what they do (function) or what they look like (phenotype). In unpublished work from our lab, we further applied reverse phenotyping to identify tumor‐reactive clonotypes. Because tumor‐reactive T cells often display dysfunctional phenotypes, reactivity markers are inherently harder to define a priori as compared to functionally preserved virus‐specific T cells. In such a context, reverse phenotyping not only yielded reactive candidate clonotypes through unbiased whole‐transcriptome shifts but also revealed which phenotypic changes tumor‐reactive clones underwent upon stimulation. Consequently, TCR sequences from tumor‐reactive clones can be readily identified, and the comparison to the unstimulated state provides an internal negative control to assess clone‐intrinsic reactivity behaviors.

Reverse phenotyping has important limitations. Because enrichment of responding cells is not possible without undermining the unbiased nature of the approach, large numbers of cells must be sequenced to reliably capture rare antigen‐reactive populations. This requirement makes reverse phenotyping experimentally demanding, costly, and impractical for routine or large‐cohort applications. In addition, the approach does not, by itself, provide a scalable solution for systematic profiling across many antigens or conditions. Reverse phenotyping is therefore best viewed as a discovery platform that generates hypotheses and candidate clonotypes for subsequent validation using more scalable approaches.

### 2.7 Sequence Similarity and TCR Deorphanization

As experimental strategies narrow the pool of candidate antigen‐reactive TCRs, computational approaches provide an additional layer of structure by exploiting sequence similarity within TCR repertoires. The central premise of these methods is that TCRs recognizing the same or closely related epitopes often share sequence features, particularly within the complementarity‐determining regions that contact pMHC complexes. By clustering TCRs based on such similarities, it becomes possible to infer shared antigen specificity even in the absence of direct experimental validation (Figure 2C).

Several frameworks have been developed to operationalize this concept, using different representations of the TCR sequence space and similarity metrics [27, 28]. Despite methodological differences, these approaches share a common goal: to group TCRs into clusters that are enriched for receptors recognizing the same epitope. Importantly, these methods do not require explicit knowledge of the cognate antigen and can therefore be applied to human repertoires at scale.

Sequence similarity‐based clustering has proven particularly powerful when combined with external reference information. When epitope specificities of TCRs are known, other TCRs with similar CDR3 sequences may recognize the same epitope. Incorporating TCRs with known epitope specificity—either from public databases or from experimentally validated datasets—thus provides critical context for interpreting clusters and for assigning putative specificities [52, 53]. In this way, known TCR‐epitope pairs act as anchors that enable inference of specificity for related, previously uncharacterized receptors. Such integration highlights that sequence‐based inference is most informative when embedded within a broader experimental framework rather than applied in isolation.

At the same time, important limitations must be acknowledged. Sequence similarity is an imperfect proxy for antigen specificity, and clustering approaches inevitably produce both false positives and false negatives—particularly when clustering relies on a single chain (TCR α or TCR β) rather than paired sequences. TCRs recognizing distinct epitopes can share sequence features, while conversely, structurally diverse TCRs may converge on the same specificity. As a result, cluster membership should be interpreted probabilistically rather than deterministically, and inferred specificities require experimental validation whenever possible [54]. In our recent work on SARS‐CoV‐2‐specific CD8^+^ and CD4^+^ T cells, we provide TCRdist [55] landscapes with “TCR anchors” of known epitope specificity, either through pHLA multimers or TCR re‐expression, illustrating both the specificity and the limitations of TCR similarity‐based clustering [52, 56].

To complicate things further, TCR cross‐reactivity is substantial. It has been estimated that one TCR may recognize up to a million different epitopes [24] (Figure 1). Mapping the recognition of dozens of TCRs against altered peptide ligands with single amino acid exchanges, we could demonstrate that approximately 10%–30% of altered peptide ligands elicit more T cell activation than the “cognate” epitope [57]. However, TCR cross‐reactivity is not limited to single amino acid exchanges. Using DNA‐barcoded pHLA multimers, Amalie Bentzen and Sine Reker Hadrup systematically probed recognition of epitopes with two amino acid exchanges, thereby calculated epitope core motifs, and estimated that each TCR may recognize more than 28,000 unique targets [17]. This questions the terminology of describing TCRs as “specific” to a given epitope or antigen and instead supports the wording of TCRs being “reactive” to a target.

These considerations further underscore a broader point: the principal bottleneck in TCR deorphanization is no longer the availability of computational tools, but the availability of high‐quality training data. Prediction of antigen specificity performs well when closely related epitopes or receptors are represented in the training set, but accuracy drops sharply for epitopes not included in the training data [30]. Notably, available training datasets remain heavily skewed towards a limited set of pathogens, further constraining generalizability. Thus, sequence similarity‐based approaches are constrained less by algorithmic sophistication than by the breadth and diversity of existing TCR‐epitope datasets.

In practice, sequence‐based inference is most effective when used to organize and prioritize TCRs that have already been enriched by experimental or biological criteria. By structuring repertoire diversity into interpretable clusters, these methods reduce complexity and guide targeted experimental validation. In the next section, I discuss how recent advances have broadened the scope of such approaches beyond qualitative specificity assignment towards quantitative prediction of TCR function.

### 2.8 Quantitative Prediction of TCR Function

Sequence similarity‐based clustering provides a qualitative framework for organizing TCR repertoires, but an important next step is the prediction of quantitative functional properties of individual receptors. In recent work, we asked whether TCR sequence information can be used not only to infer antigen specificity, but also to predict quantitative aspects of T cell responses, such as the strength of activation or functional potency [58]. When multiple TCRs recognizing the same epitope are available, or when closely related epitopes are represented, sequence‐based models can achieve meaningful levels of accuracy. For example, using 15 TCRs specific for the murine epitope H2K^b^–SIINFEKL, we predicted reactivity against altered peptide ligands (single amino acid mutants) with ~90% AUROC (true‐ vs. false‐positive rate). This level of accuracy is sufficient for prioritization, but not for deterministic classification of TCR‐epitope recognition.

These findings reinforce a central theme emerging from both experimental and computational work: the feasibility of predicting TCR function is limited less by methodological shortcomings than by the scope and balance of available data. Datasets are heavily skewed towards a small number of well‐studied pathogens and epitopes that are particularly immunodominant, while large portions of the clinically relevant antigen space remain sparsely sampled. For example, the Immune Epitope Database (www.iedb.org) contains 2326 unique publications (PMID) on HLA‐I or HLA‐II epitopes from 227 human‐pathogenic viruses overall, as of March 2026. However, there are > 150 publications on T cell assays for only six viral pathogens (Epstein–Barr virus (EBV), CMV, Influenza‐A, Hepatitis C and B virus (HCV, HBV) and Severe Acute Respiratory Syndrome Coronavirus 2 (SARS‐CoV‐2)), while the vast majority of pathogens are covered by only one publication. 2.6% of viruses thereby account for 83.6% of publications (Figure 1).

As a result, predictive models implicitly inherit these biases, performing well in familiar contexts but poorly when confronted with novel specificities. Importantly, predictive models may further reinforce this bias, as accurate predictions preferentially generate additional data within the same narrow epitope space. A critical and often underappreciated limitation is the scarcity of high‐quality negative data. For example, the absence of pHLA multimer binding does not necessarily indicate true lack of reactivity, as multimer staining can underestimate low‐affinity interactions or fail under suboptimal conditions. Similarly, antigen‐specific T cells may fail to react to antigen in stimulation assays when the cellular phenotypes limit reactivity (e.g., in case of T cell exhaustion). As a consequence, “negative” labels are often uncertain. At the same time, systematic validation of true negatives is experimentally challenging and less incentivized, as studies are typically driven by the identification of positive specificities. This imbalance results in datasets that are strongly enriched for positive examples, limiting the ability of computational models to learn discriminative features and contributing to overconfident predictions in poorly sampled regions of epitope space.

Taken together, current approaches demonstrate that quantitative prediction of TCR function is achievable within constrained and well‐characterized settings but remains unreliable at a global scale. Predictive models should therefore guide, not replace, experimental validation and functional characterization of TCRs. The next section returns to the experimental domain, focusing on how large‐scale re‐expression of candidate TCRs can be used to validate and extend computational predictions.

### 2.9 Large‐Scale TCR Re‐Expression as a Bridge From Identification to Function

As experimental and computational strategies increasingly converge on candidate sets of antigen‐reactive TCRs, a critical next step is functional validation. Large‐scale TCR re‐expression provides a direct means to bridge identification and prediction with experimentally measurable T cell behavior. Rather than relying solely on inferred specificity or sequence similarity, re‐expression enables controlled testing of defined receptors in a shared cellular background, thereby establishing causal links between TCR identity and function [32, 33, 36, 56, 57, 59].

Recent methodological advances now allow parallel re‐expression of very large numbers of TCRs, enabling the generation of highly diverse, yet experimentally tractable, T cell populations. Conceptually, this approach occupies a unique position between single‐receptor studies and bulk repertoire analyses. On the one hand, individual TCRs can be interrogated in isolation or in defined mixtures; on the other hand, populations of re‐expressed receptors can be compared under identical experimental conditions, allowing relative functional differences to emerge. Importantly, such systems make it possible to test not only whether a TCR recognizes a given antigen [32, 60, 61], but also how strongly and in what qualitative manner it responds [36, 56, 57, 59, 62]. Commercial gene synthesis costs can be as low as ~0.13 USD per nucleotide, corresponding to ~240 USD for a 1.8 kb TCR construct. This makes testing dozens of TCRs per project feasible within reasonable budgets.

In pioneering work, the groups of Ton Schumacher and Catherine Wu used transgenic TCR re‐expression to investigate tumor antigen reactivity in human tumor‐infiltrating lymphocytes (TILs), testing 104 [32] and 131 [63] to 561 [33] TCRs, respectively. In our own work, we recently re‐expressed 104 TCRs specific for two immunogenic SARS‐CoV‐2 epitopes in order to investigate the connection of TCR avidity and clonal expansion in humans [56]. The 104 TCRs were initially identified by binding to DNA‐barcoded pHLA multimers in scRNAseq, and we could validate epitope reactivity after transgenic TCR re‐expression for 98 of them (94%). Subsequent TCR‐epitope sensitivity profiling demonstrated that high, but not maximum, TCR avidity determines recruitment into memory responses, while maintenance of polyclonality ensures robustness to counteract mutational escape. These findings extended concepts previously established in mouse models, for which we re‐expressed 33 TCRs specific for SIINFEKL [57]. In a separate project, we previously tested 51 CMV‐specific human TCRs to interrogate functional aspects introduced through different ways of TCR engineering [64].

Such testing of dozens of TCRs enabled unique insights into TCR and T cell biology that were not conceivable only 10 years ago. However, the costs of several hundred USD per commercially synthesized TCR gene product still limit the number of TCRs that can be tested in a given project. In 2020, Spindler et al. [65] therefore set out to clone TCRs directly from droplet microfluidics, combining Vα‐ and Vβ‐specific primers and downstream Gibson assembly. While this enabled the generation of TCR libraries with more than 2.9 million paired TCRs, the libraries are present in bulk, so there is no direct control over individual TCRs. Moravec et al. [66] therefore developed an alternative approach that enabled them to synthesize > 1500 TCRs at material costs as low as 1 USD per TCR. In this approach, only short CDR3 regions and α chains are synthesized, and combined with pre‐existing Vα/Vβ and constant region modules [67]. Most recently, Messemaker et al. [61] and Gaglione et al. [60] refined this methodology to re‐express > 12,000 or even > 30,000 transgenic TCRs, respectively. For simple epitope reactivity screening, such numbers can be easily reached with scRNA‐seq (e.g., by testing tens of thousands of clones for binding to DNA‐barcoded pMHC multimers). However, the unique advantage of such hyper‐scale TCR re‐expression is that functional analysis can be performed with TCR‐transgenic T cells, such as target cell killing or in vivo screens. Furthermore, such synthetic polyclonal populations allow functional assays that more closely resemble physiological competition than single‐receptor experiments, while still retaining experimental control over TCR identity. TCR biology can now increasingly be interrogated at a scale that approximates the natural polyclonality of immune responses.

Large‐scale TCR re‐expression highlights an important shift in how TCR specificity and function are interrogated. Rather than asking whether a given receptor binds a particular epitope, these approaches emphasize comparative performance across many receptors under standardized conditions. This perspective naturally leads to questions about how receptor expression levels, signaling dynamics, and cellular context influence observed functional differences. Addressing these questions requires engineering strategies that restore physiological control of TCR expression, a topic that is addressed in the next chapter.

## 3 Engineering TCRs to Establish Causality

### 3.1 Why TCR Engineering Is Necessary: From Correlation to Causality

The identification of antigen‐reactive TCRs, even when supported by high‐dimensional phenotyping and functional prioritization, remains correlative. Observational analyses of TCR repertoires can reveal associations between receptor sequences, cellular states, and disease contexts, but they cannot establish whether a given TCR is causally responsible for the observed T cell behavior. Disentangling intrinsic receptor properties from extrinsic influences requires experimental perturbation.

**FIGURE 3:**
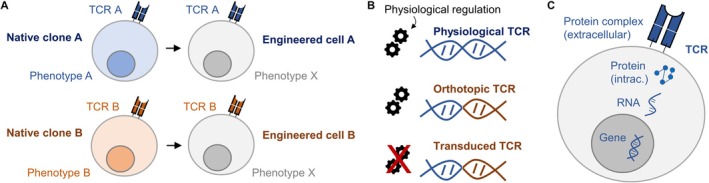
TCR engineering and orthotopic TCR replacement. (A) In native clones, expression of a given TCR leads to phenotypic imprinting. Transgenic re‐expression of TCRs in unrelated T cells enables decoupling of the TCR from the clonotypic phenotype to study TCR‐intrinsic features. (B) Orthotopic TCR replacement leads to physiologically regulated transgene expression, unlike, for example, viral TCR transduction (endogenous gene shown in blue, transgene in brown). (C) The “central dogma of molecular biology” (gene—RNA—protein) axis remains poorly characterized for the TCR, especially in dynamic settings after antigenic stimulation.

TCR engineering provides a direct means to establish such causality. By re‐expressing defined receptors in a defined cellular background, it becomes possible to isolate the contribution of the TCR itself from confounding variables such as prior activation history, differentiation state, or microenvironmental cues (Figure 3A). This is particularly important in human systems, in which longitudinal access to the same clonotype across multiple contexts is rarely possible, and genetic and environmental heterogeneity further complicate inference from observational data.

At the same time, the act of engineering itself introduces new variables that can profoundly influence T cell behavior [68]. Conventional approaches based on viral transduction [69] typically result in non‐physiological receptor expression, variable copy numbers, and altered regulatory control [70, 71, 72, 73, 74, 75]. As a consequence, engineered T cells may display functional properties that reflect the mode of receptor expression rather than intrinsic features of the TCR [64, 76]. This limitation is conceptual as much as technical: if the goal is to understand how TCRs shape T cell biology, engineering strategies must preserve rather than override physiological regulation.

Thus, the necessity of TCR engineering is twofold. First, it enables causal interrogation of receptor function that is inaccessible to purely observational approaches. Second, it exposes the importance of how receptors are expressed and regulated, revealing that the experimental framework used to study TCRs can itself shape the conclusions drawn. These considerations motivate a shift from engineering strategies optimized for convenience or expression level towards approaches that approximate physiological control. The following sections examine how such strategies have emerged and why they are essential for both mechanistic insight and translational application.

### 3.2 Limitations of Conventional TCR Engineering Approaches

Most conventional TCR engineering strategies rely on viral transduction of exogenous TCR α and β chains into primary T cells [69]. While technically robust and widely used, these approaches introduce non‐physiological features that complicate interpretation of experimental results and limit their utility for studying intrinsic TCR biology [68].

A central limitation is *mispairing with endogenous TCR chains*. In T cells that retain their native TCRs, exogenous α and β chains can pair with endogenous counterparts, generating mixed receptors with unpredictable specificity and signaling properties [77, 78, 79]. This not only reduces the effective expression of the intended receptor but also introduces safety and interpretability concerns, particularly in translational contexts. Although strategies such as additional disulfide bonds or murine constant regions have been developed to reduce mispairing [77, 78, 79], these modifications further distance engineered receptors from physiological TCR architecture.

A second issue arises from *uncontrolled receptor expression*. Viral transduction typically results in variable copy numbers and expression levels across cells, leading to heterogeneous surface TCR density even within nominally monoclonal populations [80]. Because TCR signal strength scales with receptor abundance, such variability introduces a confounding factor that is difficult to disentangle from intrinsic properties of the receptor itself [64]. Comparisons between different TCRs therefore risk reflecting differences in expression rather than intrinsic receptor properties.

Conventional engineering approaches also disrupt *endogenous regulatory control* of the TCR locus. Transduced receptors are expressed under artificial promoters and are decoupled from endogenous transcriptional and post‐transcriptional regulation. As a consequence, engineered T cells may fail to recapitulate key aspects of receptor regulation, including dynamic modulation of surface expression following antigen encounter [73, 75]. This limitation is particularly relevant when studying processes such as activation‐induced downregulation, tonic signaling, or long‐term persistence [81].

Finally, these non‐physiological features complicate efforts to relate findings from engineered systems back to in vivo T cell behavior. Functional phenotypes observed after conventional engineering may reflect artifacts of expression level or promoter choice rather than intrinsic properties of the TCR [75, 80, 82]. While such approaches remain valuable for proof‐of‐concept studies and therapeutic exploration, they are poorly suited for dissecting subtle differences in TCR signaling or for studying how receptor identity shapes long‐term fate decisions. If the goal is to understand how TCRs function in their native context, engineering strategies must move beyond convenience and address the regulatory architecture of the TCR itself. This realization has motivated the development of orthotopic replacement strategies, which integrate engineered receptors into the endogenous TCR locus to restore physiological control.

### 3.3 Orthotopic TCR Replacement of Antigen Receptors: Restoring Physiological Regulation

A major shift in antigen receptor engineering occurred when the focus moved from efficient expression towards physiological regulation. A milestone in this direction was achieved by Justin Eyquem and Michel Sadelain, who demonstrated that a CAR could be precisely inserted into the endogenous TCR alpha constant (TRAC) locus [80]. This work established the technical feasibility of targeted receptor knock‐in and also demonstrated superior in vivo performance compared to conventional transduction. Importantly, this improvement was attributed not to changes in CAR design, but to the restoration of endogenous regulatory control over receptor expression.

Building on this advance, subsequent studies extended orthotopic TCR replacement (OTR) strategies from CARs to TCRs. Work by Theodor Roth and Alex Marson demonstrated that defined TCRs could be introduced into endogenous TCR loci using targeted genome editing, enabling controlled expression of antigen‐specific receptors under physiological transcriptional regulation [83] (Figure 3B). These studies established OTR as a generalizable strategy for receptor engineering. Since then, protocol improvements have substantially elevated non‐viral knock‐in frequencies [84, 85].

In parallel, colleagues and I sought to address a limitation of earlier knock‐in approaches: the persistence of endogenous TCR chains and the resulting risk of mispairing. In work led by Thomas Müller, Dirk Busch, and myself, we demonstrated that dual‐chain editing of both TCR α and β chains prevents mispairing and yields monoclonal T cell products with defined specificity [76]. This strategy preserved the conceptual advantages of OTR while eliminating a major confounding factor of single‐chain editing.

A key insight emerging from these studies was that OTR does more than standardize receptor expression. By placing receptors under endogenous control, physiological regulation of receptor abundance is retained [76]. In our experiments, T cells carrying orthotopically replaced TCRs exhibited activation‐induced downregulation of the receptor following antigen stimulation, closely mirroring the behavior of endogenous TCRs [76]. In contrast, TCRs introduced by conventional transduction remained stably expressed at high levels even after stimulation, revealing a fundamental difference in regulation. In a follow‐up study, we demonstrated for a more expanded set of TCRs that OTR crucially improved the predictability of cell product function in vitro and in vivo [64]. These findings recapitulated earlier observations made in the context of CAR knock‐in strategies [80] and underscored that receptor regulation and cell fate are emergent properties of genomic context rather than receptor structure alone.

Together, these developments established OTR as more than a technical refinement. They revealed that how and where a receptor is expressed fundamentally shapes T cell behavior.

### 3.4 Gene–RNA–Protein Dynamics of the TCR: An Unresolved Regulatory Layer

OTR has highlighted how physiological control of TCR expression shapes T cell behavior. At the same time, it has brought into focus a major unresolved question: how regulation of the TCR is coordinated across gene, RNA, and protein levels, particularly under dynamic conditions such as antigen encounter (Figure 3C). While individual layers of this regulatory cascade have been studied extensively, they are rarely interrogated side by side, and their integration remains poorly understood.

Classical work has established that TCR surface expression is dynamically regulated at the protein level following stimulation, including rapid internalization and degradation [86]. These processes are often interpreted as negative feedback mechanisms that tune signaling strength and protect against overstimulation [86]. However, how these protein‐level changes relate to transcriptional activity at the TCR loci, transcript abundance, or RNA stability remains largely unexplored. As a result, it is unclear to what extent observed changes in TCR surface expression reflect altered gene expression, post‐transcriptional control, protein turnover, or combinations thereof [87]. A notable exception is work from the group of Jamie Cate, describing that a burst in eIF3‐dependent *TCRA* and *TCRB* mRNA translation is necessary for T cell activation [88]. Addressing these unresolved issues will require experimental platforms that integrate measurements across regulatory layers and across time. Ideally, such approaches would combine OTR‐based control of receptor expression with longitudinal profiling of transcriptional, RNA, and protein dynamics before and after antigen encounter. At present, such comprehensive analyses remain technically challenging and have been implemented only sporadically. Nonetheless, they represent a critical next step for understanding how TCR regulation contributes to functional diversity, persistence, and fate decisions in T cells.

In this context, OTR should be viewed not as a solution to TCR regulation, but as a tool that renders previously hidden regulatory processes experimentally accessible. These approaches expose the complexity of gene–RNA–protein coupling and underscore how much remains to be learned about the mechanisms that govern TCR expression and signaling under physiological conditions. This complexity becomes particularly apparent when receptors coexist in competitive settings, which is discussed in the following section.

### 3.5 CAR–TCR Cross‐Talk

CAR T cells have demonstrated clinical efficacy across a range of malignant and autoimmune indications [89, 90]. In most current manufacturing protocols, CAR expression is introduced by viral transduction, whereas the endogenous TCR remains unmodified. This permits clonotypic tracking of infused cells in patients and has revealed associations between differentiation state at infusion and long‐term in vivo persistence. Others and we have observed that CAR T cell clones displaying a more cytotoxic phenotype within the infusion product were more likely to be detected after adoptive transfer into patients [91, 92, 93, 94, 95]. However, interpretation of these observations is complicated by the highly synthetic nature of the infusion product, which consists of T cells that have undergone extensive in vitro expansion. Physiological memory formation is thought to arise from quiescent stem‐like T cells [96]. Accordingly, stem‐like populations present in the leukapheresis product ex vivo—but largely absent after in vitro expansion—have been linked to in vivo persistence and clinical response [97, 98, 99]. Notably, we observed that recruitment of CAR T cell clones from the infusion product into transcriptionally reactive states upon in vitro stimulation did not predict enhanced post‐infusion detection [95]. Strongly reactive clones may be more susceptible to activation‐induced cell death after transfer, whereas less reactive clones may persist precisely because of their relative functional restraint.

Beyond its use as a molecular barcode, it remains unclear whether the endogenous TCR actively contributes to CAR T cell function after transfer. Elimination of the endogenous TCR is often pursued to mitigate graft‐versus‐host disease in allogeneic settings [100, 101, 102]. While TCR‐deficient CAR T cells exhibit largely preserved functionality in vitro [100], in vivo observations are more complex. Reduced persistence of TCR‐deficient CAR T cells in xenograft models has been attributed to the absence of tonic TCR‐mediated signals, suggesting that endogenous receptor activity may positively influence maintenance under certain conditions [100]. Accordingly, CAR T cell products derived from T cells with predefined endogenous specificities, such as those recognizing latent viral antigens like EBV or CMV, have been explored as a means to enhance persistence through continuous antigen exposure, albeit with mixed clinical outcomes [103, 104].

Experimental studies addressing simultaneous CAR and TCR engagement have reported divergent effects depending on receptor strength and antigen abundance [105, 106, 107, 108]. The lab of Naomi Taylor observed that weak TCR stimulation attenuated CAR‐driven responses, whereas stronger signals enhance CAR function [105]. Notably, this analysis focused on target cells co‐expressing both CAR and TCR antigens, which may not reflect clinical situations in which CAR and TCR antigens are presented on distinct cell populations. Furthermore, many such studies rely on transduction of a model TCR while leaving the endogenous TCR intact. This further complicates interpretation and sets experimental systems apart from physiological settings.

In our own work, we applied a CAR–OTR system to interrogate how physiological TCR regulation modulates CAR‐driven responses under defined conditions [95]. In this system, we transduced the CAR and orthotopically replaced the endogenous TCR with a defined TCR, yielding a product that mirrors clinical CAR T cells, but with experimentally controlled TCR identity. We found that engagement of the endogenous TCR can influence CAR signaling, but importantly, the direction and magnitude of this effect depend on variables such as receptor affinity and the spatial context of antigen presentation. When TCR and CAR antigens are presented *in cis* on the same target cell, TCR engagement is additive to CAR signaling. In contrast, when CAR and TCR antigens are presented *in trans* on distinct cell populations, TCR signaling impaired CAR‐mediated cytotoxicity [95]. Receptor cross‐talk is therefore shaped not only by intrinsic signaling pathways but also by the spatial distribution of antigen. Importantly, these interactions are highly context dependent. Work from the group of Mirjam Heemskerk has demonstrated reciprocal effects, including CAR‐mediated dampening of TCR responses when TCR antigen density is low [108].

The primary value of the CAR–OTR platform lies in its ability to systematically probe receptor integration under controlled and physiologically relevant conditions. By stabilizing TCR expression through orthotopic replacement, the platform reduces expression variability and allows defined perturbations to be introduced sequentially, enabling dissection of how multiple antigen receptors integrate and compete for signals within the same cell.

## 4 TCR‐Driven Fate Decisions In Vivo

### 4.1 From the Recruitment of Single Cells to Population Dynamics After Immunization

While engineering approaches enable causal interrogation of TCR function under controlled conditions, the ultimate test of TCR biology lies in vivo, where T cells compete, adapt, and persist within complex and dynamic environments. In such settings, TCRs operate not as isolated signaling modules, but as components of a broader cell population system shaped by antigen availability, tissue context, and interactions with other cells. Understanding how TCR identity and quality influence T cell fate in vivo therefore requires integration of receptor‐level properties with population‐level dynamics.

A central challenge in this context is that in vivo T cell responses are robust as a population, but heterogeneous on a clonal level [109, 110, 111]. For any given antigen, multiple T cell clones with distinct TCRs can be recruited, expanded, or lost over time. These clones may differ in activation kinetics, differentiation trajectories, and long‐term persistence, even when recognizing the same epitope. As a result, population‐level immune responses often mask substantial inter‐clonal, and even intra‐clonal, diversity at the single‐cell level.

Importantly, the in vivo fate of T cells cannot be inferred solely from early activation events. Clonal hierarchies evolve over time, shaped by factors such as repeated antigen exposure, competition for resources, and migration between anatomical sites. Subtle TCR‐dependent differences early on may become amplified during prolonged responses, leading to dominance or extinction of individual clonotypes [112]. Conversely, early stochastic events can have lasting consequences for clonal composition, complicating attempts to predict long‐term outcomes from initial measurements [109, 110, 113].

Much of our current understanding of TCR‐driven fate decisions has been derived from mouse model systems, which have provided critical insights into clonal recruitment, expansion, and memory formation [113, 114, 115]. These studies established key principles, including diverse naïve precursor pools and the probabilistic nature of single‐cell fate decisions. However, translating these principles to human immunity remains challenging, owing to differences in antigen exposure history, repertoire diversity, and experimental accessibility [3, 116, 117].

Recent advances now enable to revisit these questions in human systems with unprecedented resolution. High‐throughput TCR sequencing, single‐cell profiling, and spatially resolved analyses allow longitudinal tracking of clonotypes across tissues and time [118]. These approaches provide a framework to examine how TCR identity contributes to population responses in vivo—not as an abstract property, but as a measurable determinant of clonal behavior within complex immune responses. The following sections build on this framework to dissect the mechanisms that govern recruitment, diversity, persistence, and spatial organization of T cell clonotypes in vivo.

### 4.2 Naïve Precursor Populations and Recruitment Into Immune Responses

**FIGURE 4:**
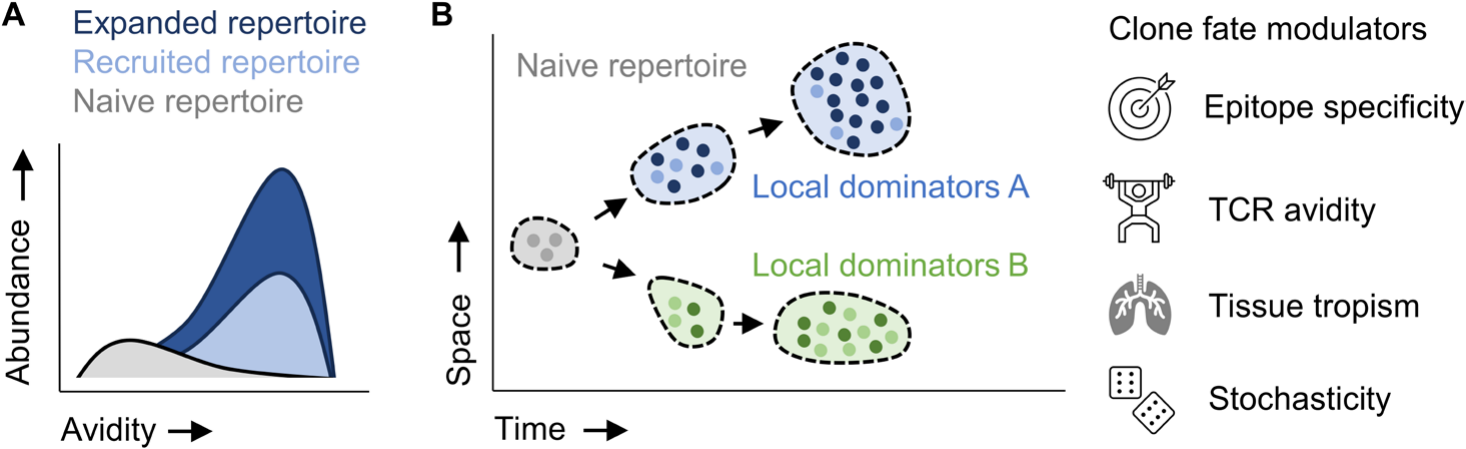
TCR dependent clonal fate in vivo. (A) Most precursor clones in the naïve repertoire are of low avidity towards a given epitope. Antigen exposure leads to preferential recruitment of clones with a minimum threshold of TCR avidity. Beyond this threshold, however, fine differences among generally high TCR avidity values do not dictate clonal expansion. (B) Clonal T cell fate in vivo develops along a spatiotemporal trajectory, which can be influenced by epitope specificity, TCR avidity, tissue tropism or stochasticity.

Any T cell response begins with recruitment from the naïve T cell pool (Figure 4A). For a given epitope, this pool consists of a finite number of naïve precursors whose TCRs can recognize the antigen with sufficient functional avidity to cross the activation threshold. Pioneering work from the groups of Leo Lefrançois and Mark Jenkins in mouse models established that these precursor populations are small but measurable for CD8^+^ and CD4^+^ T cells, typically comprising tens to hundreds of cells per epitope, and Nicole La Gruta and Stephen Turner demonstrated that they are highly diverse at the level of TCR sequence [119, 120, 121]. For the murine model epitope H2K^b^/SIINFEKL, we could ourselves refine the measurement of the average precursor frequency to 351 naïve T cells per entire mouse, with each TCR represented only once [57]. This number equates to a frequency of 3 × 10^−5^ of total CD8^+^ T cells. These studies provided the first quantitative framework for understanding how many cells are available to seed an immune response and how repertoire diversity is distributed before antigen encounter.

A key insight from this body of work is that naïve precursor populations are not uniform, which is also the case in humans [122, 123]. Seminal work by Alanio et al. [123] demonstrated that, for the same pathogen, average precursor frequencies for different epitopes can differ by up to an order of magnitude (10‐fold). Generally, precursor frequencies in humans seem to lie in the range of 5 × 10^−7^ to 1 × 10^−5^ of total CD8^+^ T cells. Even for a single epitope, precursor cells differ in TCR sequence, signaling sensitivity, and activation potential [124, 125, 126]. As a result, recruitment into an immune response is probabilistic at the single‐cell level: some precursors are activated rapidly, others more slowly or not at all [57]. This stochasticity reflects both intrinsic receptor properties and extrinsic factors such as antigen dose, presentation kinetics, and competition within secondary lymphoid organs [111]. Of note, it is accepted also in the B cell field that stochasticity is not just noise, but an inherent biological principle ensuring clonal diversification [127, 128].

Despite this single‐cell variability, immune responses at the population level are remarkably robust. The diversity of the naïve precursor pool introduces redundancy, such that loss of individual clones does not compromise effective immunity [56, 57]. In other words, multiple related clonotypes contributing to a T cell response buffer stochastic single‐cell effects and enable reproducible population‐level outcomes. This principle helps to reconcile how immune responses can be both variable at the cellular level and consistent across individuals [113].

The size and composition of naïve precursor pools also impose constraints on recruitment thresholds. TCRs must exceed a minimal level of functional avidity to participate in a response, but above this threshold, fine‐grained differences in receptor strength do not necessarily translate into proportional differences in clonal expansion [56, 57]. In the murine model of *Listeria monocytogenes* ‐OVA (expressing the model epitope SIINFEKL) infection, we could demonstrate that there is a clear correlation between clonal expansion and TCR avidity when considering the entire avidity spectrum of clones from a naïve repertoire. This naïve repertoire spanned several orders of magnitude in EC_50_ values (epitope peptide doses of 10^−4^ to 10^−11^ M eliciting half‐maximum T cell activation). It thereby also encompassed clones with ultra‐low avidities that were clearly antigen‐reactive, but did not reach the technical limit of detection for functional avidity measurement (here 10^−4^ M) [57]. Concomitantly, clones had a higher likelihood of being activated (indicated by CD44 positivity) the higher their avidity was. However, among clones with generally “high” avidity (10^−10^ to 10^−11^ M), the correlation between individual TCR avidity and clonal expansion or recruitment probability was lost. In this case, “high” avidity refers to clones from the naïve repertoire with the highest order of EC_50_ value magnitude (where EC_50_ values differ by less than one order of magnitude) [57], which corresponds to the avidity range of clones we previously retrieved from an antigen‐experienced memory response against the same epitope H2K^b^/SIINFEKL [59]. Thus, large differences in TCR avidity matter for recruitment and expansion, whereas smaller differences may be averaged out across the responding population.

These principles are established in experimental models but their relevance to human immunity has been difficult to assess directly. Recently, we re‐expressed 104 TCRs specific for the SARS‐CoV‐2 epitopes HLA‐A*01:01/LTD or HLA‐A*02:01/YLQ identified from repertoires of five vaccinated donors using scRNAseq and TCR sequencing (TCRseq) [56]. OTR allowed interrogation of TCR‐intrinsic functional avidities of these SARS‐CoV‐2‐specific TCRs, and correlation with in vivo clonal expansion determined through scRNA/TCRseq. Fine differences in TCR avidity within the high‐avidity range did not correlate with clonal expansion. What first seemed counterintuitive is actually consistent with our previous murine studies outlined above [57]. Consistently, we observed that the only two TCRs with a markedly lower avidity (with EC50 values several orders of magnitude below the remaining TCRs) were “locked” in a naïve or naïve‐like state and did not clonally expand. In the following sections, we build on this framework to examine how polyclonality, competition, and temporal evolution further shape T cell responses after recruitment.

### 4.3 Polyclonality, Redundancy, and the Emergence of Deterministic Immune Responses

Following recruitment from the naïve precursor pool, T cell responses typically evolve as polyclonal populations rather than expansions of single dominant clonotypes. Even when a response is directed against a single epitope, multiple TCRs with distinct sequences and signaling properties are recruited and expanded. This polyclonality is a direct consequence of the diversity present in the naïve repertoire and represents a fundamental organizational principle of adaptive immunity.

Elegant single‐cell transfer and lineage‐tracing experiments in mouse models demonstrated that individual naïve T cells responding to the same antigen can give rise to markedly different progeny (e.g., up to 100,000‐fold difference in progeny size), even when activated under ostensibly identical conditions [109, 110]. Consistently, the same public clone can generate very different responses both in terms of clonal expansion and in terms of phenotype in different mice as part of an endogenous response [111]. These studies revealed that clonal expansion, differentiation into effector or memory subsets, and long‐term persistence are highly variable at the single‐cell level. It is worth stressing that endogenous T cell responses (as typically tracked in humans and often also in mice) derive from polyclonal precursors where each TCR is present only in a single cell. Each clonotype participating in an endogenous response therefore effectively represents a single‐cell–derived lineage, with the associated variability in expansion and fate.

The relationship between stochastic single‐cell behavior and reproducible population‐level outcomes can be illustrated by a simple analogy. Tossing die once yields an unpredictable result: the outcome is inherently stochastic, and no meaningful prediction can be made. However, tossing the same die one hundred times produces a highly reproducible distribution, with approximately one sixth of outcomes per face. Importantly, the underlying process has not become deterministic; rather, stochastic events are averaged across repeated trials, yielding a predictable population‐level behavior.

T cell responses operate according to a similar logic. The activation and fate of an individual naïve T cell responding to antigen are difficult to predict, reflecting stochastic influences at the single‐cell level [129]. However, when many naïve precursors participate in a response, the aggregate behavior of the population becomes robust and reproducible. Polyclonality and redundancy thus act as mechanisms that transform stochastic cellular decisions into consistent immune outcomes. For example, 2 out of 18 H2K^b^/SIINFEKL‐specific clones (i.e., 11.1%) that we identified from a naïve repertoire and characterized functionally, showed an avidity as high as one typically observes in antigen‐experienced populations [57]. Extrapolating this number to the total number of precursor cells we identified in an entire mouse (*n* = 351), this equates to 39 high‐avidity T cells. While this extrapolation is approximate, it is interesting to note that Buchholz et al. [110] determined that the successful transfer of 28 cells (100 cell‐transfer with 28% take‐rate) suffices to yield robust population dynamics as opposed to variable single‐cell derived responses. Thus, while the fate of any single cell remains unpredictable, a redundancy of only a few dozen high‐avidity clones is sufficient for adaptive immunity to operate robustly.

Within this framework, differences in TCR properties can be understood as modulating probabilities rather than dictating outcomes. Loading a die—for example, by increasing the likelihood of rolling a particular number—does not eliminate randomness, but it shifts the distribution of outcomes. Analogously, properties such as TCR avidity bias the likelihood that a given clonotype will be recruited, expand, or persist, without rendering its fate deterministic (Figure 4A). High‐avidity TCRs increase the probability of favorable outcomes, particularly at early stages of recruitment, but they do not guarantee dominance or long‐term success at the level of individual clones. The groups of Dirk Busch and Veit Buchholz illustrated this point using OT‐II CD4^+^ T cells and altered peptide ligands with different avidities. While single‐cell derived primary responses were highly variable in terms of progeny size and phenotype, secondary responses from the same populations became predictable [112].

This probabilistic framework reconciles seemingly conflicting observations in T cell biology: variability on the single‐cell and robustness on the population level. In the next section, we extend this framework to consider how probabilities are reshaped over time, as clonal competition and selection progressively sculpt the T cell repertoire in vivo.

### 4.4 Temporal Evolution, Clonal Competition, and Repertoire Reshaping

Once recruited, T cell responses do not remain static. Instead, clonal composition evolves over time as responding T cells proliferate, differentiate, migrate, and are selectively maintained or lost. This temporal dimension adds an additional layer of complexity to TCR‐driven fate decisions, as early stochastic differences between clones can be amplified, dampened, or reversed as immune responses progress.

During the expansion phase, clonal competition emerges as a key organizing force. Responding T cells compete for access to antigen, costimulatory signals, cytokines, and physical niches within lymphoid and peripheral tissues. Importantly, these resources are neither uniformly distributed nor static over time. As antigen availability declines or tissue environments change, selective pressures acting on clonotypes shift accordingly. Clones that were initially minor contributors can gain relative advantage at later stages, while early dominant clones may contract disproportionately. Consistently, Aoki et al. [130] or we in Kocher et al. [56] found that clones that are prominent after primary SARS‐CoV‐2 vaccination in humans are not correlated with clonal abundance after secondary vaccination (Figure 4A). While stochastic features may shape each step of a longitudinal response, selective pressures operating over time can generate emergent patterns that appear deterministic at later stages—a form of intra‐host repertoire evolution.

For example, using polyclonal adoptive transfers of TCRs with different avidities against H2K^b^/SIINFEKL, we showed that low‐avidity clones are not dominant during the acute phase, but can emerge as the dominant clones during latency of murine CMV engineered to express OVA [59]. Importantly, such late dominance does not contradict early recruitment thresholds, but reflects shifting selective pressures during chronic antigen exposure. Even when using the same TCR (i.e., OT‐I), Grassmann et al. [131] demonstrated that T cell clones that show so‐called inflationary responses during latent murine CMV infection are not necessarily highly expanded during the acute phase of infection. The size of inflationary responses was better predicted by the content of stem‐like cells in the acute phase, thereby identifying these cells as *bona fide* memory precursors. In recent work, we could show—using yellow‐fever vaccination in humans next to mouse models—that such stem‐like memory precursors are characterized by metabolic quiescence [126].

From a TCR‐centric perspective, this temporal plasticity complicates attempts to assign fixed functional rankings to receptors. A TCR that confers an early expansion advantage may not be optimal for long‐term persistence, tissue residency, or recall responses. Conversely, receptors that confer modest early expansion may favor survival or functional maintenance under chronic or low‐level antigen exposure. Thus, TCR‐dependent effects must be interpreted in the context of when they are assessed during an immune response and what the immunological context is (acute versus chronic infection, latent persistence, vaccination, or tumor immunity).

These dynamics have important implications for both mechanistic studies and therapeutic applications. Observations made at single time points risk capturing transient states rather than stable properties of clonotypes. Longitudinal analyses are therefore essential to distinguish early stochastic effects from sustained selective advantages. In the following section, we return to the role of TCR avidity within this temporal framework, clarifying when receptor‐intrinsic differences matter, when they are averaged out, and under which conditions they become decisive.

### 4.5 TCR Avidity, Functional Heterogeneity, and the Limits of Peripheral Biomarkers

The role of TCR avidity in shaping T cell responses is frequently misunderstood. A common intuition is that higher avidity receptors should uniformly confer superior functional outcomes. While this view has intuitive appeal, it does not hold up once probabilistic and population‐level principles are considered.

At the level of recruitment, large differences in TCR avidity matter. Receptors must exceed a minimal functional threshold to participate in an immune response, and TCRs below this threshold are unlikely to be recruited [56, 57]. In this sense, avidity acts as a gatekeeper. However, once this threshold is crossed, finer differences between intermediate and high‐avidity receptors often have limited predictive power for population‐level outcomes. In polyclonal responses seeded by many naïve precursors, such differences are effectively averaged out, consistent with the probabilistic framework discussed earlier.

This logic may lead to the conclusion that TCR avidity is largely irrelevant beyond recruitment. Such an interpretation, however, conflates population‐level robustness with single‐clone behavior. While small avidity differences may not strongly influence the aggregate response of a polyclonal population, they can become decisive when stochastic buffering through averaging effects is removed. This distinction becomes particularly apparent in settings when monoclonal populations with high cell numbers are transferred, as often done in experimental mouse models, but also for adoptive cell therapies in humans.

In our own work, we analyzed T cell responses against defined neo‐epitopes in blood from patients with melanoma. Clonal abundance in blood did not correlate with functional avidity [36]. In the same study, we used a MC38‐OVA tumor mouse model and transferred monoclonal populations of TCRs with different avidities, with sufficient cell numbers per population (e.g., 500 cells) to make “TCR‐deterministic” behavior visible beyond mere stochasticity effects. In an infection model with *L.m*.‐OVA, the exact same TCRs followed the intuitive pattern of higher avidity coinciding with higher clonal expansion. In the MC38‐OVA tumor model, however, clonal abundance did not correlate with functional avidity, neither in blood nor in the tumors. Intriguingly, instead of clonal abundance, two other features strongly correlated with TCR avidity in the tumor model: mouse survival and PD‐1 expression levels in peripheral blood (but not in the tumor, where PD‐1 expression was uniformly high) [36]. These results demonstrated that differences in TCR avidity (i) do not necessarily lead to expected patterns of clonal abundance since chronic antigen exposure can mix up clonal hierarchies, (ii) have a deterministic effect for survival, likely since high transferred cell numbers override stochastic effects; (iii) lead to more likely and/or efficient T cell priming, resulting in stratification of different PD‐1 expression levels, which were overall intermediate, reflecting co‐inhibition as a natural feedback mechanism to T cell activation [132]; and (iv) do not translate into differentially high levels of PD‐1 expression in the tumor, where PD‐1 expression levels are overall higher, reflecting T cell exhaustion and suggesting that environmental features (e.g., abundance of antigen) can again override “deterministic” features mediated through the TCR.

Of note, our study did not establish a new marker of tumor reactivity per se—this has been demonstrated previously by multiple groups for markers such as PD‐1, CD39, or CXCL13 [133, 134, 135]. Rather, we determined that differences in peripheral phenotypes reflect quantitative differences in intrinsic T cell functionality. Instead of serving as binary indicators of reactivity, those other markers may also reflect quantitative differences in underlying TCR‐driven functionality. Understanding this distinction is essential for translating biomarker‐based selection strategies into rational therapeutic design.

In terms of tumor control, the MC38‐OVA model we used was—as many tumor models in preclinical studies—not well reflective of human tumorigenesis, which happens more successively and over a much longer period of time. Although mice were followed for up to 80 days, one may argue that our model still represented more of an “acute‐phase response”. Along these lines, the jury is still out whether lower TCR avidities may have a competitive advantage during long‐term antigen exposure (reviewed by us previously [136]), similar to what we have seen during latent CMV infection [59]. It is tempting to speculate that lower TCR avidity (and/or TCR downregulation) may protect from T cell exhaustion [75, 137], although the evidence for this in humans remains limited. Indirect evidence that mild, not maximum receptor signaling is compatible with superior in vivo outcomes in the context of human T cells was rather generated for CAR T cells [138, 139]. This ambiguity has led to the conception that perhaps there is a sweet spot (or “Goldilocks zone”) of TCR avidity, which balances out the advantages and disadvantages of too‐high or too‐low TCR avidity [137, 140].

Overall, TCR avidity can appear dispensable in some settings, yet critical in others. In endogenous immune responses, redundancy and competition buffer against fine‐grained differences between individual TCRs. In engineered or therapeutic contexts, particularly those involving monoclonal or oligoclonal cell products, the same differences can dominate outcomes. The functional importance of avidity is not absolute, but conditional on the structure of the responding population.

Together, these considerations emphasize that TCR avidity shapes immune responses by biasing probabilities rather than dictating outcomes. Its effects are constrained by redundancy in physiological settings but become unmasked when clonal diversity is reduced. Recognizing this context dependence is essential for interpreting TCR function in vivo and for designing engineered T cell therapies that rely on defined receptor populations.

### 4.6 Spatial Organization of TCR Clonotypes and Context‐Dependent Fate Decisions

T cell fate decisions are not determined by receptor properties alone but unfold within specific anatomical and cellular contexts. Where a T cell encounters antigen—within secondary lymphoid organs, peripheral tissues, or specialized microenvironments—profoundly shapes activation dynamics, differentiation trajectories, and long‐term persistence [141]. Intriguingly, for example, human lymph nodes and the spleen harbor minimally overlapping TCR repertoires [142]. Spatial organization therefore represents a critical but historically underappreciated dimension of TCR‐driven immune responses.

Recent advances in spatially resolved transcriptomics and in situ TCR identification have begun to illuminate how clonotypes are distributed across tissues and niches [143, 144, 145]. These approaches now make it possible to localize individual T cell clones within intact tissue architecture and to link receptor identity to local transcriptional states. Such analyses reveal that clonotypes recognizing the same epitope need not be spatially homogeneous. Instead, distinct clones can occupy different niches, reflecting differences in activation history, specialization, or antigen access.

Spatial context is particularly relevant during priming, where antigen presentation is temporally and anatomically constrained [146]. Elegant recent work in mice has demonstrated that the timing and location of T cell interaction with antigen‐presenting cells influence subsequent differentiation and memory formation. In tumor‐draining lymph nodes, sustained rather than prematurely interrupted TCR signaling led to expansion of stem‐like TCF‐1^+^ SLAMF6^high^ T cells [147]. This was made possible through expression of PD‐1, which was disproportionately expressed in high‐affinity T cells, preventing precocious terminal differentiation of high‐avidity clones. Within this framework, TCR avidity biases—but does not dictate—the likelihood of productive antigen encounters. High‐avidity receptors may increase the probability of stable interactions under limiting antigen conditions, whereas lower‐avidity receptors may require more favorable spatial or temporal alignment to be recruited. As with other aspects of TCR biology, avidity shifts probabilities rather than it imposes deterministic outcomes.

Beyond priming, spatial segregation can shape clonal competition and functional specialization in peripheral tissues (Figure 4B). Tumors, inflamed organs, or mucosal sites present heterogeneous landscapes in which antigen density, cytokine gradients, and cellular composition vary locally. In such environments, the same clonotype may adopt different functional states depending on its precise location, while different clonotypes that recognize the same antigen may be differentially retained or excluded from key niches. For example, a recent study by the group of Laura Mackay used the TCR as a barcode to study clonal overlap between tissue‐resident (T_RM_) and exhausted (T_EX_) T cells in human breast cancer and revealed that these two lineages were largely non‐overlapping [148]. Similarly, colonic *ITGB2* ^+^ T_EM_‐like cells and *ITGAE* ^+^ T_RM_‐like cells seem to represent different lineages in human checkpoint inhibitor‐induced colitis based on non‐overlapping TCR usage [149].

Secondary lymphoid organs offer a particularly tractable setting to study these principles in humans. Tonsils represent accessible lymphoid tissue in which antigen exposure, T cell priming, and differentiation can be examined in situ. Lisa Wagar and colleagues have developed an elegant computational approach to simulate clonal mixing between tonsils and blood to demonstrate disproportionate compartmentalization of individual clones [150]. Emerging studies leveraging tonsillar tissue have revealed striking spatial organization of T cell subsets and provide a window into human priming dynamics that complements insights from experimental models [151]. For example, the team around “Slide‐TCR‐seq” showed that clones within and outside of germinal centers are different, validating their findings statistically also through an “observed versus shuffled” comparison [145]. Integrating spatial information with TCR identity in such settings has the potential to reveal how receptor properties intersect with anatomical context to shape immune responses, but the field is just at the beginning of applying such methodological possibilities to biological questions.

Taken together, spatial analysis adds a critical dimension to our understanding of TCR‐driven fate decisions. It reinforces the view that T cell responses emerge from the interplay of receptor‐intrinsic properties, probabilistic recruitment, competition, and environmental context (Figure 4B). By situating TCR clonotypes within the tissues and niches in which they operate, spatial approaches offer a means to connect molecular specificity with biological outcome in vivo. These insights also underscore why understanding TCR biology requires moving beyond linear models and embracing multidimensional frameworks that integrate sequence, function, time, and space.

## 5 Outlook

Over the past decade, the study of TCRs has undergone a qualitative shift. What was once a field constrained by technical limitations is now defined by the challenge of integrating vast amounts of high‐dimensional data across molecular, cellular, and population scales. Advances in TCR identification, physiological engineering, and in vivo fate mapping have transformed what can be measured. The central question moving forward is no longer whether TCR biology can be interrogated at scale, but how these measurements can be integrated into coherent biological principles.

A recurring theme throughout this review is that TCR‐driven immune responses are inherently probabilistic. Single‐cell fate decisions are shaped by stochastic events, while robustness emerges at the population level through redundancy, competition, and spatial organization. Properties such as TCR avidity bias probabilities rather than dictate outcomes, and their importance depends on context, scale, and time. Recognizing this probabilistic architecture is essential for reconciling apparently conflicting observations and for avoiding overly deterministic interpretations of TCR function.

Physiological TCR engineering has emerged as a critical tool. Rather than serving solely as a therapeutic strategy, approaches such as OTR enable causal interrogation of TCR biology under controlled yet biologically relevant conditions. By restoring endogenous regulation, these strategies expose regulatory layers, including gene–RNA–protein coupling and receptor cross‐talk, that are obscured by conventional overexpression systems. At the same time, they highlight how incomplete our understanding of TCR regulation remains, particularly under dynamic conditions of antigen exposure.

A major limitation of our current knowledge is the uneven coverage of the antigenic space. While TCRs specific for a small number of pathogens and model epitopes have been studied extensively, large fractions of the clinically relevant antigen space remain poorly characterized. This imbalance constrains both mechanistic insight and predictive modeling. Expanding TCR–epitope datasets in a manner that reflects real‐world disease prevalence, rather than historical or technical convenience, represents a critical task for the field. Without such data, even sophisticated computational approaches will remain inherently constrained.

The growing ability to profile TCR repertoires at scale raises important questions of clinical responsibility. Repertoire‐based diagnostics and immune monitoring have the potential to provide unprecedented insight into immune history and disease state, but they also carry risks of overinterpretation and unintended clinical consequences. Realizing their promise will require clearly defined use cases, rigorous validation, and thoughtful integration into healthcare systems.

Human immunity poses both the greatest challenge and the greatest opportunity. Experimental model systems have been indispensable for establishing core principles of T cell biology and will remain essential for mechanistic dissection. However, translating these principles to humans requires direct study of human T cell responses, with all their inherent heterogeneity, exposure history, and spatial complexity. Emerging approaches that integrate single‐cell profiling, spatial analysis, and longitudinal sampling in human tissues—including secondary lymphoid organs—offer promising avenues to bridge this gap. Ultimately, understanding TCR biology will require embracing its probabilistic, context‐dependent, and multidimensional nature—integrating sequence, regulation, time, and space into a unified framework.

## Funding

This work was supported by Bundesministerium für Forschung, Technologie und Raumfahrt (01KI2015), Deutsche Forschungsgemeinschaft (401821119, SCHO 1949/1‐1), Horizon Europe Programme (No. 101137459), Aventis Foundation, project 2024: Life Science Bridge Award.

## Conflicts of Interest

The author declares no conflicts of interest.

## Data Availability

Data sharing not applicable to this article as no datasets were generated or analyzed during the current study.
